# Assignment of EC Numbers to Enzymatic Reactions with Reaction Difference Fingerprints

**DOI:** 10.1371/journal.pone.0052901

**Published:** 2012-12-28

**Authors:** Qian-Nan Hu, Hui Zhu, Xiaobing Li, Manman Zhang, Zhe Deng, Xiaoyan Yang, Zixin Deng

**Affiliations:** Key Laboratory of Combinatorial Biosynthesis and Drug Discovery (Wuhan University), Ministry of Education, and Wuhan University School of Pharmaceutical Sciences, Wuhan, P. R. China; University of Westminster, United Kingdom

## Abstract

The EC numbers represent enzymes and enzyme genes (genomic information), but they are also utilized as identifiers of enzymatic reactions (chemical information). In the present work (ECAssigner), our newly proposed reaction difference fingerprints (RDF) are applied to assign EC numbers to enzymatic reactions. The fingerprints of reactant molecules minus the fingerprints of product molecules will generate reaction difference fingerprints, which are then used to calculate reaction Euclidean distance, a reaction similarity measurement, of two reactions. The EC number of the most similar training reaction will be assigned to an input reaction. For 5120 balanced enzymatic reactions, the RDF with a fingerprint length at 3 obtained at the sub-subclass, subclass, and main class level with cross-validation accuracies of 83.1%, 86.7%, and 92.6% respectively. Compared with three published methods, ECAssigner is the first fully automatic server for EC number assignment. The EC assignment system (ECAssigner) is freely available via: http://cadd.whu.edu.cn/ecassigner/.

## Introduction

The Enzyme Commission number (EC number) is a numerical classification scheme for enzymes, based on the chemical reactions they catalyze [1]. Strictly speaking, EC numbers do not specify enzymes, but enzyme-catalyzed reactions. There are 6 main EC levels in the EC system: (1) EC 1 for Oxidoreductase reactions, (2) EC 2 for Transferase reactions, (3) EC 3 for Hydrolase reactions, (4) EC 4 for Lyase reactions, (5) EC 5 for Isomerase reactions, and (6) EC 6 for Ligase reactions. The basis of linking genomics and chemistry is the EC numbers (1).

A wide range of research areas in molecular biology and medical biochemistry require a reliable enzyme classification system, *e.g.*, metabolic network reconstruction and systems biology. When research scientists in the above mentioned areas wish to unambiguously refer to an enzyme and its function, the EC number introduced by the Nomenclature Committee of the International Union of Biochemistry and Molecular Biology (IUBMB) is used [2].

The EC numbers represent enzymatic reactions (chemical information), but they are also utilized as identifiers of enzymes and enzyme genes (genomic information) [3]. The EC number plays a key role in classifying enzymatic reactions and in linking the enzyme genes or proteins to reactions in metabolic pathways [4]. This duality of the EC numbers makes it possible to link the genomic repertoires of enzyme genes to the chemical repertoire of metabolic pathways [3].

The assignment of the EC numbers is performed manually, based on published experimental data on individual enzymes, by the Joint Commission on Biochemical Nomenclature of the International Union of Biochemistry and Molecular Biology and the International Union of Pure and Applied Chemistry. Unfortunately, there are numerous reactions known to be present in various pathways but without any official EC numbers, because the EC number assignment requires published articles on full characterization of enzymes [3], [4].

There are some computational EC assignment systems, which are purely based on chemical knowledge, without any use of protein sequence or other information on enzymes [3]–[6].

In E-zyme [4], the authors proposed a ‘RDM (reaction center (R), the difference region (D), and the matched region (M)) patterns’ method to predict the potential EC numbers to given reactant pairs (substrates and products) or uncharacterized reactions. The method consists of three steps: (i) graph alignment of a query reactant pair (substrates and products) for computing the query RDM pattern, (ii) multi-layered partial template matching by comparing the query RDM pattern with template patterns related with known EC numbers and (iii) weighted major voting scheme for selecting appropriate EC numbers. What should be noted here is that the input of E-zyme is a query reactant pair (substrates and products). For whole reactions, E-zyme is based on a manual RPAIR (reactant pairs) database [7], [8]. Therefore, in the E-zyme server, the method is currently only applicable to two molecules by using a graph comparison method [9].

Latino *et al*. [6] developed a method using MOLMAP (molecular mapping of atom-level properties) descriptors, which relies on a Kohonen SOM (self-organizing map) that defines types of covalent bonds on the basis of their physicochemical and topological properties. The MOLMAP descriptor of a molecule represents the types of bonds available in that molecule. The MOLMAP descriptor of a reaction is defined as the difference between the MOLMAPs of the products and the reactants and numerically encodes the pattern of changes in bonds during a chemical reaction. A genome-scale data set of enzymatic reactions available in the KEGG (Kyoto Encyclopedia of Genes and Genomes) database was encoded by the MOLMAP descriptors and was explored for the assignment of the official EC number from the reaction equation with Random Forests as the machine learning algorithm. In the method (using MOLMAP), physicochemical descriptors [10] are used to describe reactions. The descriptors are very helpful to understand reaction mechanisms [11], [12], however limited by the commercial availability.

Egelhofer *et al.* [2] have developed tools for the validation of the EC number classification scheme, in which there are two major steps: (1) the chemical similarity calculation using atom and bond types, and (2) the characterization of the individual reaction by considering reaction pairs. The authors defined at least one and if necessary more than one unique difference keys for each sub-subclass of the EC number classification system. In the method, the authors only considered atom or bond types.

Similarity concept is widely used in biological and chemical studies. Automatic perception of similarities between metabolic reactions, *i.e.* their classification, is a Chemoinformatics issue with an impact in bioinformatics, biotechnology, or medicinal chemistry [6], [12]. There are some researchers developing different methods to measure reaction similarity between individual steps of enzymatic reaction mechanisms and to quantitatively measure the similarity of enzymatic reactions based upon their explicit mechanisms [13], [14]. Ridder and Wagener [15] clustered a data set of metabolic reactions using a difference fingerprint defined by the differences in occurrences of each SYBYL atom type in the reactant and product fingerprints. Faulon *et al* [16] employed molecular signatures of topological atom neighborhoods to derive reaction signatures and used such descriptors with SVM (support vector machine) to classify metabolic reactions in terms of EC numbers. In RxnFinder [17], our group proposed reaction difference fingerprints and reaction similarity to search biochemical reactions. In this work, reaction difference fingerprints, which not only consider atom and bond types, but also bond connections (variable path lengths), are applied to assign EC numbers to enzymatic reactions.

## Methods

### Reaction Selection

The 5120 training reactions used in ECAssigner are selected from 8458 KEGG [18] reactions after excluding the following six cases (some cases are overlapped):

1. 965 reactions with missing molecules (unbalanced reactions) (for example, NH_3_+ CO_2_< = > Cyanide (R00152)),
2. 395 reactions with Glycans (such as Glycan+H_2_O< = >D-Mannose+Glycan (R01331)),
3. 1280 reactions without EC assignments, for instance,
  - (3a) a non-enzymatic reaction, 2 D-Urobilinogen+Oxygen < = >2 D-Urobilin +2 H2O (R00070);
  - (3b) an incomplete reaction, Protein< = >L-Alanyl-tRNA (R00165);
4. 932 reactions with molecules having not structure information, for example, 16 ATP +16 H_2_O +8 Reduced ferredoxin < = >8 e- +16 Orthophosphate+16 ADP +8 Oxidized ferredoxin (R00002),
5. 1174 reactions with R-atom types or polymer-like molecules,
  - (5a) a reaction example for R-atoms, 2 Long-chain alcohol+Oxygen < = >2 Long-chain aldehyde +2 H2O (R00041);
  - (5b) a reaction example for polymer-like molecules, Polyphosphate+n H2O< = >(n+1) Oligophosphate (R00001),
6. 99 reactions (61 reactions are racemase or epimerase reactions and 38 reactions having zero fingerprints when fingerprint length is selected at 3) with zero reaction difference fingerprints, for instance, L-Glutamate< = >D-Glutamate (R00260) glutamate racemase.

### Molecular Fingerprints

The molecular fingerprint (MFP) of a molecule is defined as MFP = (F_i_), in which F_i_ refers to a molecular fragment with real occurrences of a molecule. F_i_ is obtained by molecular fragmentation method. Here, we use the linear fragments up to 7 atoms (6 bonds) that are computed by OpenBabel package [19].

### Reaction Difference Fingerprints

The fingerprints of reactant molecules minus the fingerprints of product molecules will generate reaction difference fingerprints (RFP) defined as: RFP = MFP _reactants_- MFP _products_ = (RF_i_), where RF_i_ refers to a difference fragment with difference occurrences of reactants and products. For KEGG reaction Urea-1-carboxylate+H_2_O < = >2 CO_2_+2 NH_3_ (R00005: C01010+ C00001< = >2 C00011+2 C00014), the reaction difference fingerprints are calculated using formula RFP_ R00005_ = MFP _C01010+C00001_− MFP _2C00011+2C00014_.

### Euclidean Distance

After calculating reaction difference fingerprints of two reactions, the similarity of two reactions can be computed using a Euclidean distance measurement as defined as D_i,j_ = ED(RFP_ i_, RFP_ j_). The significance of the distance values will be discussed in the RESULTS AND DISCUSSIONS section.

### Assignment Strategy

The smaller the distance between two reactions, the more similar they are. The EC number of the closest (the most similar), with minimum distance, training reaction will be assigned to an input reaction. If there are several reactions having the same minimum distance, the EC number occurring most often will be selected.

## Results and Discussion

### Length Selection of Reaction Difference Fingerprints

**Figure 1:**
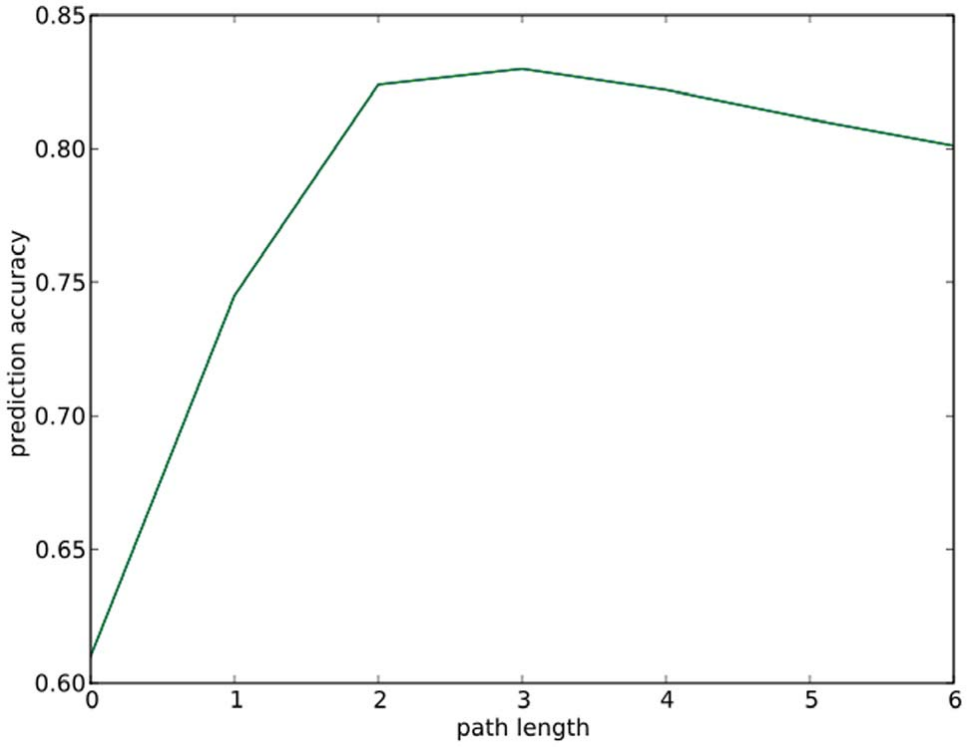
Cross-validation accuracies of reaction difference fingerprints with different lengths.

In the work, the first step is to select the fingerprint length. In the model development, different fragment lengths are used to get leave-one-out cross-validation (as applied by Yamanisi *et al*. [4]) accuracies as shown in Table 1, which are plotted in Figure 1. From the path length-accuracy plot, the model using fragments with path length equal to 3 is the best. In ECAssigner, path length is selected as 3.

**Table 1:** Cross-validation accuracies of reaction difference fingerprints with different lengths.

Length	sub-sub-class	sub-class	main
0	0.614	0.671	0.856
0–1	0.742	0.785	0.901
0–2	0.822	0.859	0.923
0–3	0.831	0.867	0.926
0–4	0.826	0.860	0.918
0–5	0.817	0.849	0.907
0–6	0.807	0.840	0.903

With fingerprint length increases from 0 to 6, the cross-validation accuracies on sub-sub-class, sub-class and main class will increase until the length reaches 3, then the accuracies will decrease.

### Performance of the Selected Reaction Difference Fingerprints

The validation accuracy is 0.831; 0.867, 0.926 for sub-subclass, subclass, main class respectively. Then, the validation accuracy is investigated on the individual EC levels as shown in Table 2, from which the rank order of accuracy is: EC2>EC3>EC1>EC6>EC4>EC5. More than 82% (2112+1398+735)/5120 reactions are with EC 1, 2, or 3. The results on main classes EC1, 2, and 3 are better than those on EC4, 5, and 6.

**Table 2:** Cross-validation accuracy performance over different EC levels.

Totalreactions,precision	EC1reactions,precision	EC2reactions,precision	EC3reactions,precision	EC4reactions,precision	EC5reactions,precision	EC6reactions,precision
5120 0.831	2112 0.828	1398 0.880	735 0.861	532 0.748	190 0.657	153 0.784

With a selected fingerprint length at 3, the number of reactions and the cross-validation accuracies will vary from EC1 to EC6.

### Results Over Reaction Distances

**Figure 2:**
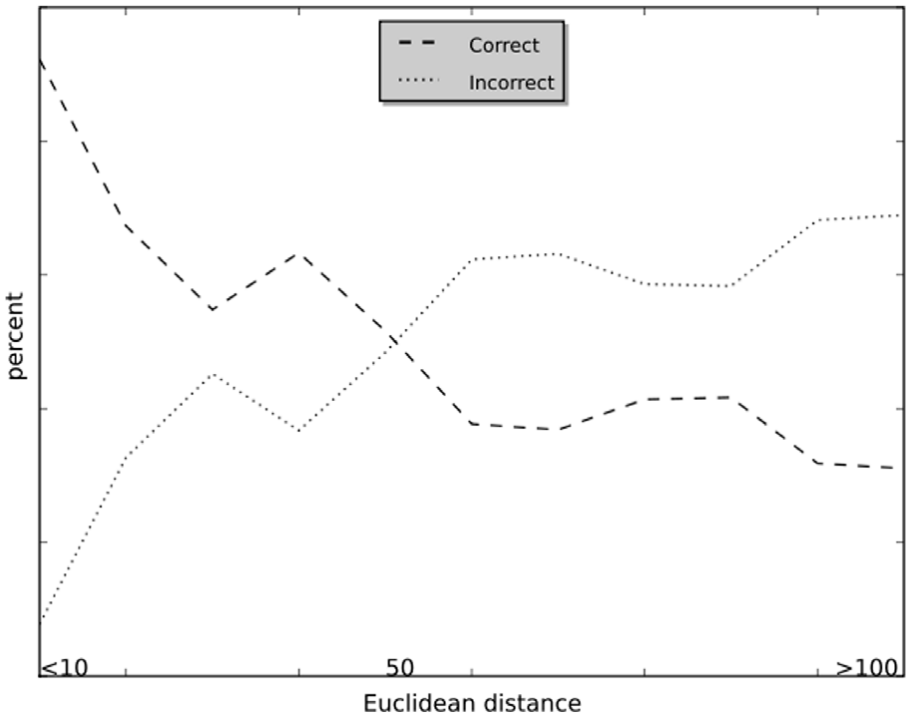
Correct and incorrect results made over different reaction distances.

EC number is assigned according to the Euclidean distance of the input reaction to the training reactions. The validated results are counted with the distance to the closest training reaction as shown in Figure 2. From Figure 2, 85% reactions are predicted with distance smaller than 20, in which more than 90% predictions are correct. From the cross-validation results, the accuracy will become worse with the increase of Euclidean distance. The predictions made with distances smaller than 50 will lead to higher accuracy.

### Incorrect Assignments

Two main reasons for the incorrect assignments are analyzed: (1) the same chemical transformation pattern with different participating molecules; (2) the same chemical transformation pattern with intra-molecular and inter-molecular difference.

**Figure 3:**
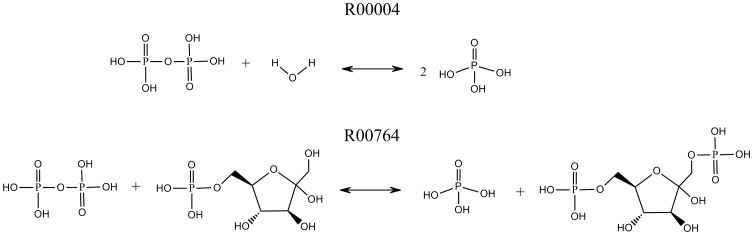
Incorrect prediction KEGG reaction examples on different substrate specificities.

For the first case, a reaction Diphosphate+H_2_O < = > 2 Orthophosphate (R00004) by another reaction Diphosphate+D-Fructose 6-phosphate < = > Orthophosphate+D-Fructose 1,6-bisphosphate (R00764) (as shown in Fig. 3), R00004 is a diphosphate phosphohydrolase or pyrophosphate phosphohydrolase reaction. The EC number of R00004 is 3.6.1. The closest reaction to R00004 is R00764, which is a phosphotransferase reaction. The EC number of R00764 is 2.7.1. Both reactions cleave an O-P bond and add an oxygen atom to a Phosphate atom. The difference is that R00004 will use water (3.6.1) and R00764 will use a molecule other than water (2.7.1).

**Figure 4:**
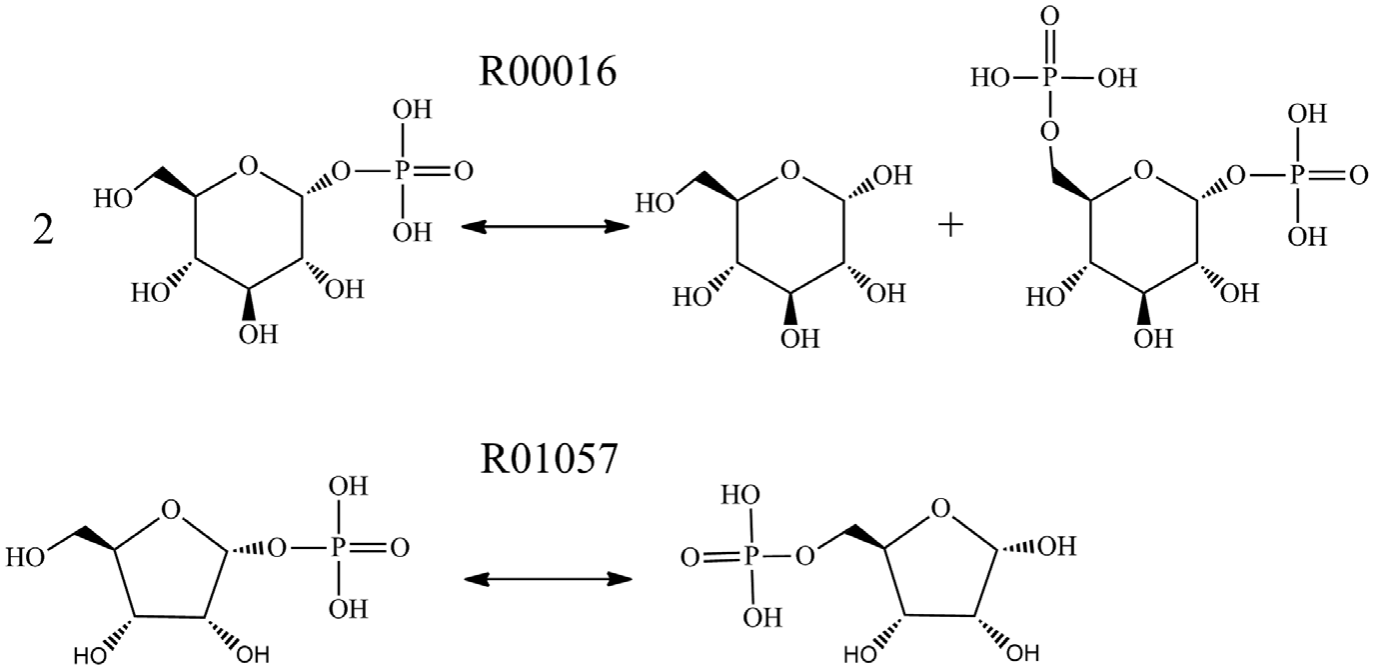
Incorrect prediction KEGG reaction examples on inter-molecular and intra-molecular transformations.

For the second reason, a reaction D-glucose-1-phosphate 6-phosphotransferase (R00016) by another reaction D-Ribose 1,5-phosphomutase (R01057) (as shown in Fig. 4), R00016 belongs to 2.7.1 and R01057 is classified as 5.4.2. Both reactions transfer phosphate group however in different ways. R00016 is an inter-molecular transformation; however R01057 is an intra-molecular reaction.

### EC Number Assignments of those Reactions without Experimental Evidence

**Figure 5:**
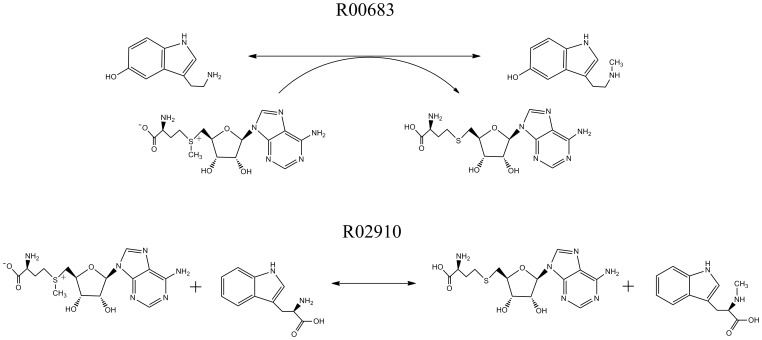
One KEGG reaction example, S-Adenosyl-L-methionine+L-Tryptophan< = >S-Adenosyl-L-homocysteine+Abrine (R00683, ‘enzyme not yet characterized’), and its closest training KEGG reaction, S-Adenosyl-L-methionine+Serotonin< = >S-Adenosyl-L-homocysteine+N-Methylserotonin (R02910), for EC assignment prediction.

An important application is to assign EC numbers for those reactions without enzyme validations. We applied our method to the first reaction example commented as ‘enzyme not yet characterized’, that is, S-Adenosyl-L-methionine+L-Tryptophan < = > S-Adenosyl-L-homocysteine+Abrine (R00683) (as shown in Fig. 5). There are three steps in the EC number assignments:

1. calculate the RDF of the input reaction, for instance, RFP_ R00683_ for R00683,
2. compute distances to the training reactions and obtain the minimum distance. For R00683, the minimum is 2, which relates with S-Adenosyl-L-methionine+Serotonin< = >S-Adenosyl-L-homocysteine+N-Methylserotonin (R02910) (as shown in Fig. 5),
3. assign the EC number of the training reaction corresponding with minimum distance to the input reaction. The EC number of R02910 is 2.1.1. The EC number of R00683 is thus assigned as 2.1.1, which is commented as ‘Transferases. Transferring one-carbon groups. Methyltransferases’.

From the comparisons of R00683 and R02910, the EC number assignment is reasonable, which indicates that ECAssigner could be potentially applied to predict a reference EC number for a query reaction.

### Comparisons with Other Methods

Comparisons with EC number assignment methods are listed in Table 3. E-zyme [4] applied an error-prone manual method (2) to assign EC numbers for whole reactions. Based on manually curated RPAIR, E-zyme is not an automatic server for a whole reaction. However, E-zyme applied automatic method for two molecules (one reactant and one product). The method explored by Latino *et al.* [6], used physicochemical properties calculated by commercial software. Egelhofer *et al.* [2] computes difference keys (atom or bond types) in their strategy, however they only considered the reaction difference of atom and bond types. Both Latino *et al.* [6] and Egelhofer *et al.* [2] didn’t provide web server for their methods. From the comparisons, ECAssigner is the first automatic server for EC number assignment.

**Table 3:** Comparisons of several EC assignment methods by considering their method basis, if they are automatic for a whole reaction, and if there is a web server available.

Method	Method Basis	Automatic for Whole Reaction	Online Server
Yamanisi *et al*., 2009	RPAIR (manual)	No	Yes for two molecules
Latino *et al.,* 2009	Petra package (commercial)	Yes	No
Egelhofer *et al.,* 2010	Difference keys (atoms or bonds)	Yes	No
ECAssigner	Reaction difference fingerprints	Yes	Yes

### Conclusions

The results demonstrate that the combination of reaction difference fingerprints (RDF) with Euclidean distance obtained satisfactory models to assign EC numbers for enzymatic reactions. The RDF descriptor of a reaction is defined as the difference between the molecular fingerprints of the products and the reactants and numerically encodes the transformation pattern of changes during a chemical reaction. A fingerprint length at 3 is validated. From the comparisons, ECAssigner calculates reaction difference fingerprints with variable lengths directly from chemical structures of a reaction, which does not need manual steps or commercial packages.
